# Tricuspid Endocarditis Leading to a Stroke: A Case Report of Paradoxical Embolism in a Young Intravenous Drug User

**DOI:** 10.7759/cureus.78304

**Published:** 2025-01-31

**Authors:** Khaleel Quasem, Michelle Carrasquel, Varun George, Lee Elisevich, Haley Maser

**Affiliations:** 1 Internal Medicine, McLaren Greater Lansing, Lansing, USA; 2 Internal Medicine, Michigan State University, East Lansing, USA; 3 Internal Medicine, Mclaren Greater Lansing, Lansing, USA; 4 Cardiology, McLaren Greater Lansing, Lansing, USA; 5 Cardiology, Michigan State University, East Lansing, USA

**Keywords:** antiplatelet therapy, cerebral infarction, infective endocarditis, intracardiac shunt, intravenous drug user, paradoxical embolism, patent foramen ovale, septic pulmonary emboli, tricuspid valve endocarditis

## Abstract

Infective endocarditis (IE) involving the tricuspid valve is commonly associated with intravenous drug use, with right-sided IE typically leading to septic pulmonary emboli; however, systemic embolization via paradoxical embolism is a rare and severe complication. We present a case of a 33-year-old female with a history of intravenous drug use who was admitted with generalized pain after leaving another facility against medical advice following treatment for pneumonia and tricuspid valve endocarditis, including vegetation debulking. On readmission, she exhibited signs of infection, and imaging revealed bilateral septic pulmonary emboli with cavitary lesions, while echocardiography showed severe tricuspid regurgitation with large mobile vegetation and an aneurysmal interatrial septum, suggesting a probable patent foramen ovale. Despite management with intravenous antibiotics for *Staphylococcus aureus* bacteremia, the patient declined surgical intervention, and on the 15th day of hospitalization, she developed acute confusion. A brain MRI demonstrated acute infarcts in the left frontal and occipital lobes and left cerebellum, indicative of cardiac embolization. Low-dose aspirin was initiated, and she was transferred for further intervention; however, she ultimately succumbed to repeated neurological embolization and severe septic shock. This case underscores the importance of echocardiographic evaluation to detect intracardiac shunts in patients with right-sided IE and highlights the critical role of timely, multidisciplinary management, including considerations for early antiplatelet therapy and surgical intervention, in preventing catastrophic complications such as paradoxical embolism and cerebral infarcts. Additionally, it reinforces the importance of addressing social determinants of health and public health strategies to mitigate the increasing burden of IE among intravenous drug users.

## Introduction

Infective endocarditis (IE) involving the tricuspid valve is commonly associated with intravenous drug use (IVDU). While right-sided IE typically results in septic pulmonary emboli, systemic embolization via paradoxical embolism is rare. This occurs when venous emboli bypass the lungs and enter systemic circulation, often through a patent foramen ovale (PFO) or other intracardiac shunts, which affect approximately 6.9 per 1,000 live births in North America [1]. Up to 30% of the general population has a PFO [2], providing a potential pathway for paradoxical embolism.

Cerebrovascular insult is the second leading cause of mortality worldwide, with 45% of cases managed without an identifiable etiology. Notably, disparities in cardiovascular outcomes contribute to worse prognoses among underserved populations, where limited access to healthcare, delayed diagnosis, and socioeconomic barriers exacerbate the disease burden [3]. Social determinants of health, including homelessness, substance use disorder, and lack of access to preventive care, play a crucial role in the outcomes of patients with IE. The patient in this case had multiple risk factors, including IVDU and limited access to healthcare, which likely contributed to the severity of her illness and her decision to decline surgical intervention.

IE occurs in approximately 15 of every 100,000 people in the United States, and its incidence has risen in recent years [4]. This increase is largely attributed to the opioid epidemic and the rising prevalence of IVDU, but additional factors include increased survival of patients with congenital heart disease, rising use of implantable cardiac devices, and increased healthcare-associated infections. Between 2000 and 2013, the proportion of IVDU-associated endocarditis hospitalizations in the United States rose from 7% to 12% [5]. These trends highlight the need for targeted public health interventions, early screening, and harm-reduction strategies to mitigate the growing burden of IE among high-risk populations.

## Case presentation

**Figure 1:**
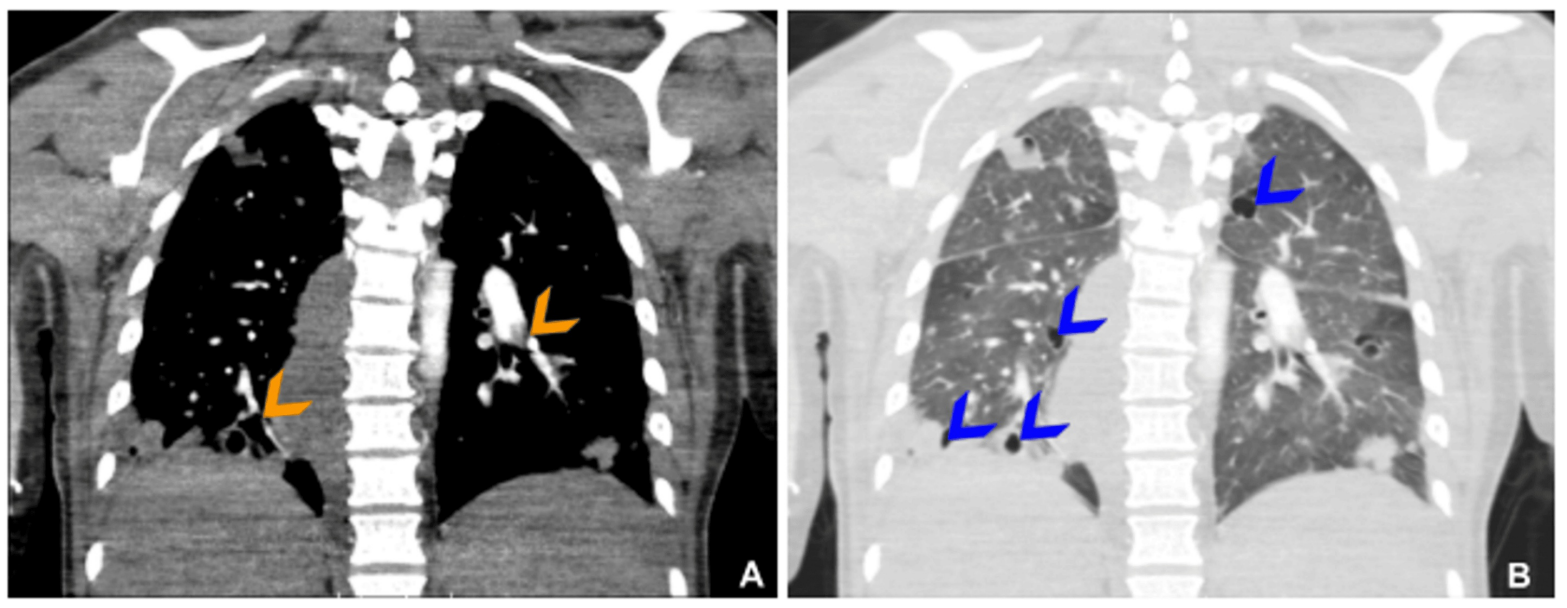
CT findings: bilateral pulmonary emboli and necrotic airspace opacities. A coronal chest CT scan revealed bilateral segmental pulmonary emboli (orange arrows, image A) without evidence of central embolus or overt right heart strain. Numerous nodular airspace opacities were seen bilaterally (blue arrows, image B), some demonstrating cystic and necrotic components, compatible with septic emboli.

**Figure 2:**
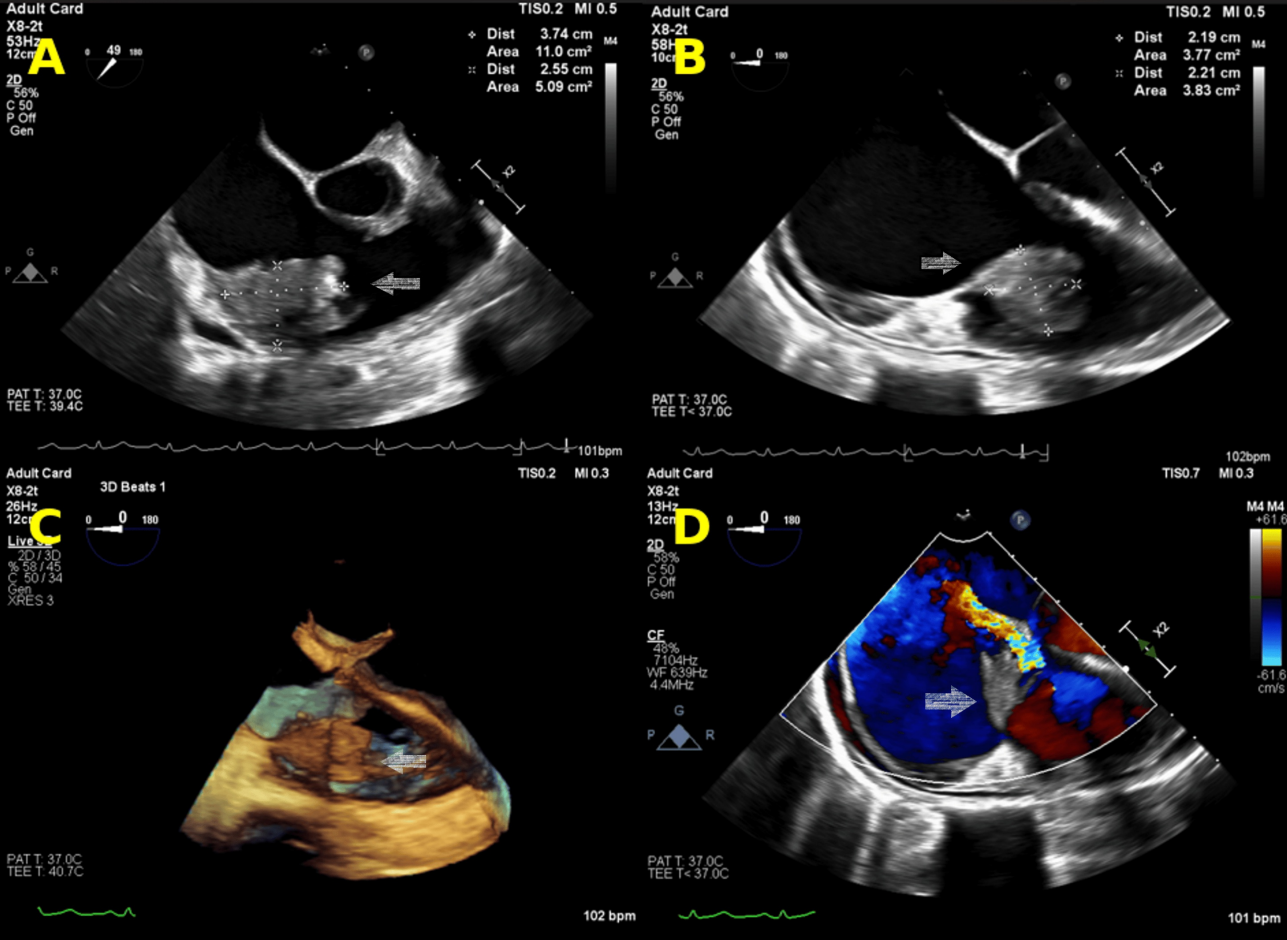
Transesophageal echocardiography (TEE) imaging of the tricuspid valve in infective endocarditis. (A) Midesophageal view at a 49° omniplane angle showing a vegetation measuring 3.7 × 2.6 cm. (B) Midesophageal view at a 0° omniplane angle with vegetation measuring 2.2 × 2.2 cm. (C) 3D echocardiographic image visualizing the large vegetation on the anterior tricuspid valve leaflet. (D) Midesophageal view with color Doppler demonstrating severe eccentric tricuspid regurgitation.

A 33-year-old female with a history of IVDU presented to the emergency department with generalized pain. One week prior, she had left against medical advice from another medical facility after treatment for pneumonia and tricuspid valve IE with vegetation debulking. On readmission, she was tachycardic and mildly hypotensive, with leukocytosis, anemia, and elevated inflammatory markers (Tables 1, 2). Pulmonary CT (Figure 1) demonstrated bilateral septic pulmonary emboli and cavitary lesions. Transthoracic echocardiography (TTE) revealed severe tricuspid regurgitation, mild right ventricular dilation with evidence of pressure overload, and a large, mobile vegetation (2.4 x 1.7 cm) attached to the posterior leaflet of the tricuspid valve. Transesophageal echocardiography (TEE) (Figure 2) confirmed these findings and identified an aneurysmal interatrial septum with probable PFO.

**Table 1:** Abnormal admission laboratory findings. This table highlights only the abnormal lab values on admission, emphasizing leukocytosis, severe anemia, electrolyte imbalances, and renal dysfunction. WBC: white blood cells; RBC: red blood cells; Hgb: hemoglobin; Hct: hematocrit: RDW: red cell distribution width; CRP: C-reactive protein; CO2: carbon dioxide; BUN: blood urea nitrogen; eGFR: estimated glomerular filtration rate; AST: aspartate aminotransferase; INR: international normalized ratio; A/G ratio: albumin/globulin ratio.

Category	Test	Value	Reference range
Hematology	WBC	13.22 x 10³/uL	4.0-10.0 x 10³/uL
	RBC	1.9 x 10⁶/uL	4.2-5.4 x 10⁶/uL
	Hgb	5.2 g/dL	12-16 g/dL
	Hct	15.2%	36-46%
	Platelets	94 x 10³/uL	150-450 x 10³/uL
	RDW	18.8%	11.5-14.5%
Inflammatory markers	Neutrophil auto %	84%	40-75%
	Lymphocyte auto %	8.2%	20-45%
	CRP	26.8 mg/dL	<3.0 mg/dL
Electrolytes	Sodium	124 mmol/L	135-145 mmol/L
	Chloride	90 mmol/L	96-106 mmol/L
	CO2	19 mmol/L	22-29 mmol/L
	Calcium	8.1 mg/dL	8.5-10.5 mg/dL
Renal function	BUN	44.7 mg/dL	7-20 mg/dL
	BUN/creatinine ratio	34.38	10-20
	eGFR	56 mL/min/1.73m²	>60 mL/min/1.73m²
Liver function	AST	52 U/L	10-40 U/L
	Alkaline phosphatase	154 U/L	40-130 U/L
	Bilirubin total	1.5 mg/dL	0.1-1.2 mg/dL
Coagulation panel	Prothrombin time	15.4 sec	11-14 sec
	INR	1.43	0.8-1.2
Metabolic panel	Anion gap	15 mmol/L	7-16 mmol/L
Protein panel	Globulin	4.7 g/dL	2.0-3.5 g/dL
	A/G ratio	0.53	1.2-2.2

**Table 2:** Summary of vital signs on admission. This table summarizes the vital signs of a 33-year-old female at the time of admission, highlighting tachycardia, mild hypotension, and normal oxygen saturation.

Vital sign	Value	Reference range
Temperature	37.8°C (oral)	36.1-37.2°C
Heart rate	124 bpm	60-100 bpm
Respiratory rate	23 breaths/min	12-20 breaths/min
Blood pressure	95/62 mmHg	90/60-120/80 mmHg
Blood pressure (manual)	105/72 mmHg	90/60-120/80 mmHg
Oxygen saturation (SpO2)	95%	95-100%
Height	163 cm	N/A
Weight	59.1 kg	N/A

Despite being offered surgical intervention, the patient declined. The exact reasons for her refusal were not documented, but potential contributing factors include fear of postoperative complications, distrust in the medical system, and social determinants such as substance use disorder and lack of support systems. She was managed conservatively with intravenous broad-spectrum antibiotics for *Staphylococcus aureus* bacteremia.

**Figure 3:**
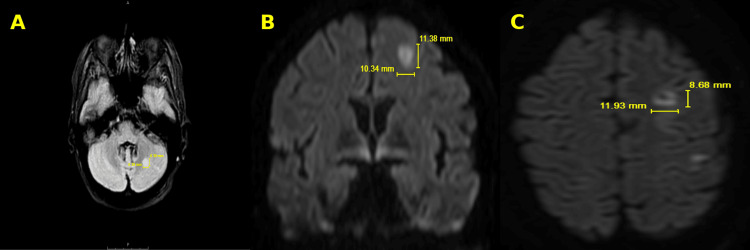
MRI brain imaging: lesion mapping with dimensions. Small focal areas of restricted diffusion in the left frontal lobe cortex near the vertex (A & B). Focal ill-defined T2 fluid-attenuated inversion recovery (FLAIR) hyperintense signal abnormalities in the left cerebellum (C).

On day 15 of hospitalization, she developed acute confusion and altered mental status. Emergent MRI (Figure 3) of the brain showed evidence of acute infarcts in the left frontal and occipital lobes and the left cerebellum, consistent with cardiac embolization. The patient was initiated on low-dose aspirin and transferred to a tertiary cardiac facility for repeated percutaneous debulking therapy and surgical tricuspid valve repair. The patient expired following transfer after repeated neurological embolization and severe septic shock.

## Discussion

Tricuspid valve endocarditis is a common complication among intravenous drug users and frequently results in septic pulmonary emboli and cavitations [5,6]. Rarely, systemic embolic events, termed paradoxical embolism, occur when venous emboli bypass the pulmonary circulation through a cardiac defect, such as a PFO or another septal deformity, allowing direct entry into the systemic circulation and increasing the risk of major end-organ complications. This embolism may then travel to the brain or other systemic arteries, leading to neurological deficits and increased morbidity.

We present an unusual complication of right-sided endocarditis-cerebral embolic infarcts due to paradoxical embolism. The presence of a large, mobile tricuspid vegetation and aneurysmal interatrial septum with probable PFO likely provided both a site for septic emboli formation as well as a pathway for right-to-left intracardiac shunting, resulting in paradoxical embolization. Identifying intracardiac shunts in right-sided endocarditis is crucial to reducing morbidity and mortality, as studies have shown that patients with untreated PFOs have up to a 50% increased risk of embolic events compared to those without structural heart disease [1,2]. Moreover, early detection and management of these anomalies can improve patient outcomes by allowing for timely risk stratification and therapeutic intervention. Echocardiography, specifically TEE, is critical in detecting such structural anomalies and guiding the management of uncorrected intracardiac shunts when indicated.

The management of paradoxical embolism in endocarditis is challenging, particularly in cases where surgical intervention is declined. Surgical interventions such as valve replacement or repair can reduce the risk of further embolic events; however, the decision must be balanced with the patient’s preferences and comorbid conditions. A meta-analysis of surgical vs. medical management in endocarditis found that early valve surgery was associated with a 40% lower risk of recurrent embolic events across a cohort of 1,200 patients, reinforcing the importance of timely intervention in select cases [6]. Additionally, patients with larger vegetations (>10 mm) have been shown to have a higher risk of embolization, further justifying surgical intervention in specific cases.

In this case, aspirin was initiated to mitigate the risk of further cerebral events following an initial cerebrovascular insult. Initiating consideration of earlier antiplatelet therapy, at the time of discovery of the PFO following TEE, may have prevented cerebrovascular accident altogether. The role of anticoagulation in tricuspid endocarditis remains controversial due to the potential risk of hemorrhagic complications; however, it may be warranted in special circumstances and requires further investigation. For instance, a systematic review of 10 clinical studies reported that IE patients receiving prior antiplatelet therapy had a 15-20% reduction in systemic embolic events compared to those who did not receive antithrombotics, with statistical significance (p < 0.05), though bleeding risk remained a concern [7]. Given these findings, further research is needed to clarify patient selection criteria for early antithrombotic therapy in IE.

Therefore, the decision to initiate antiplatelet therapy and/or surgical valve repair/replacement in IE patients with suspected intracardiac shunting necessitates timely multidisciplinary discussion, taking into account embolic risk, bleeding risk, and patient comorbidities. Future prospective studies, particularly randomized controlled trials, are warranted to further clarify the optimal timing and indications for antithrombotic therapy in this patient population, as current data remain limited to retrospective analyses and observational studies., particularly in those with large vegetations and identified intracardiac shunting anomalies.

## Conclusions

This case highlights the importance of early detection of intracardiac shunting through echocardiography, as it allows for risk stratification and potential early intervention, which may prevent catastrophic embolic events. The integration of multidisciplinary teams in managing right-sided IE is essential to optimizing patient outcomes and reducing embolic complications. While surgical interventions, such as valve repair or replacement, remain the definitive treatment to prevent recurrent embolic events, patient preferences, comorbidities, and operative risks must be carefully considered. Addressing the rising burden of IE in high-risk populations requires not only improved clinical management but also harm reduction strategies, early screening initiatives, and expanded access to addiction treatment programs.

Further research is needed to refine evidence-based guidelines on the role of early antiplatelet or anticoagulation therapy, the optimal timing of surgical intervention, and strategies to improve surgical acceptance among patients with intravenous drug use. By integrating timely diagnosis, individualized treatment strategies, and preventive care, clinicians can better address the challenges of right-sided IE and its potentially fatal complications, such as paradoxical embolism.

## References

[1] Shahjehan RD, Abraham J (2024). Intracardiac shunts. StatPearls.

[2] Hakman EN, Cowling KM (2024). Paradoxical embolism. StatPearls.

[3] Wang A, Gaca JG, Chu VH (2018). Management considerations in infective endocarditis: a review. JAMA.

[4] Wurcel AG, Anderson JE, Chui KK, Skinner S, Knox TA, Snydman DR, Stopka TJ (2016). Increasing infectious endocarditis admissions among young people who inject drugs. Open Forum Infect Dis.

[5] Chan P, Ogilby JD, Segal B (1989). Tricuspid valve endocarditis. Am Heart J.

[6] Zhang RS, Alam U, Maqsood MH (2023). Outcomes with percutaneous debulking of tricuspid valve endocarditis. Circ Cardiovasc Interv.

[7] Caldonazo T, Musleh R, Moschovas A (2024). Antithrombotic therapy in patients with infective endocarditis: a systematic review and meta-analysis. JACC Adv.

